# Identification of Avocado Fruit Disease Caused by *Diaporthe phaseolorum* and *Colletotrichum fructicola* in China

**DOI:** 10.3390/jof11080547

**Published:** 2025-07-23

**Authors:** Aosiqi Ma, Yuhang Xu, Hongxing Feng, Yanyuan Du, Huan Liu, Song Yang, Jie Chen, Xin Hao

**Affiliations:** 1 Key Laboratory of Forest Resources Conservation and Utilization in the Southwest Mountains of China, Ministry of Education, Southwest Forestry University, Kunming 650224, China; masq@swfu.edu.cn (A.M.); 13837070286@163.com (Y.X.); 15908232564@163.com (Y.D.); jiechen@swfu.edu.cn (J.C.); 2 Key Laboratory of Forest Disaster Warning and Control of Yunnan Province, Southwest Forestry University, Kunming 650224, China; 15087859119@163.com; 3 Plant Science, Wageningen University & Research, 6708 PB Wageningen, The Netherlands; huan.liu@wur.nl

**Keywords:** fruit rot, fruit anthracnose, *Persea americana*

## Abstract

*Persea americana* (avocado) is a healthy fruit, rich in unsaturated fatty acids, various minerals, and vitamins. As avocado cultivation continues to expand globally, its development is increasingly constrained by concomitant diseases, among which fruit rot and anthracnose have emerged as significant threats to fruit quality. Menglian in Yunnan Province is the largest avocado production area in China. In November 2024, fruit rot was observed on avocado fruits in Yunnan, China, characterized by reddish-brown discoloration, premature ripening, softening, and pericarp decay, with a field infection rate of 22%. Concurrently, anthracnose was detected in avocado fruits, presenting as small dark brown spots that developed into irregular rust-colored lesions, followed by dry rot depressions, ultimately leading to soft rot, peeling, or hardened dry rot, with a field infection rate of 15%. Infected fruit samples were collected, and fungal strains were isolated, purified, and inoculated via spore suspension, followed by re-isolation. The strains were conclusively identified as *Diaporthe phaseolorum* (SWFU20, SWFU21) and *Colletotrichum fructicola* (SWFU12, SWFU13) through an integrated approach combining DNA extraction, polymerase chain reaction (PCR), sequencing, phylogenetic reconstruction, and morphological characterization. This is the first report of *D. phaseolorum* causing fruit rot and *C. fructicola* causing anthracnose on avocado in China. In future research, we will test methods for the control of *D. phaseolorum* and *C. fructicola*. The identification of these pathogens provides a foundation for future disease management research, supporting the sustainable development of the avocado industry.

## 1. Introduction

Avocado (*Persea americana*), a tropical fruit renowned for its nutritional richness, features a yellow-green flesh and a distinct buttery aroma, earning it the moniker “forest butter”. Apart from being a good source of protein, fats, carbohydrates, and vitamins, avocados are also abundant in essential minerals like sodium, potassium, magnesium, and calcium [1]. Due to its exceptional taste and nutritional profile, avocados have gained popularity worldwide, emerging as a primary source of income for farmers in tropical regions [2]. However, as the cultivation of avocados expands and global consumption rises, the prevalence of diseases has emerged as a significant challenge, posing a critical constraint on the avocado industry’s progress. The primary quality issues affecting avocado fruits are flesh bruising and fruit rot [3].

Post-harvest fruit rot is a prevalent affliction of avocado fruits, primarily instigated by *Aspergillus* spp., *Botryosphaeria* spp., and other fungi [4]. These pathogens generate hyphae on fruit surfaces, instigating decay and significantly impeding fruit storage and transportation [5]. The disease manifests initially with inconspicuous symptoms pre-harvest, presenting as small, shallow lesions. However, symptoms rapidly escalate during fruit softening and ripening. Initially, small, irregular brown to red spots emerge on the fruit’s skin, with subsequent pathogen infiltration of the vascular bundle, resulting in brown stripes on the flesh. Following this, purple-brown irregular spots develop on the pedicel. As ripening progresses, the skin lesions darken, becoming sunken, the fruit shrinks, and the surface becomes coated with gray-brown mycelium and spores, culminating in decay and the release of brown juice, accompanied by a fetid odor [6]. Fruit-rot fungi have emerged as primary pathogens impacting avocado quality, inducing canker, wilt, and fruit rot. These pathogenic fungi compromise fruit quality, leading to substantial yield losses, posing a severe threat to the economic viability and sustainability of avocado cultivation. Presently, management of fruit rot disease predominantly involves the application of carbendazim, thiophanate-methyl, tebuconazole, and propiconazole during fruit development, alongside the utilization of Bordeaux mixture and stone sulfur mixture through spraying or smearing on the fruit [7].

Fruit anthracnose, caused by *Colletotrichum* sp., is a significant disease affecting avocado fruit yield and quality [8,9]. This fungal disease primarily targets mature fruits, leaves, twigs, flowers, and young fruits, leading to fruit drop and decay. Infections are more prevalent in environments with high temperatures and humidity, resulting in the development of black spots on the fruit surface, substantial fruit drop, and rot. Studies indicate that avocado fruits in various countries such as Australia, Mexico, Ghana, Brazil, and Chile have been affected by anthracnose to varying extents, with up to 90% infection rates during severe outbreaks, causing significant economic losses [10]. In China, avocado fruits have exhibited typical anthracnose symptoms during postharvest storage, with an incidence rate of 25.8% [11]. Currently, research on avocado anthracnose is limited, with a primary focus on pathogen isolation and identification. Species of the genus *Colletotrichum* are recognized internationally as comprising one of the top ten groups of plant pathogenic fungi due to their widespread presence, high destructiveness, and scientific significance as a model disease system. Notably, *C. anthrisci* [9], *C. siamense*, *C. fructicola* [12], and *C. perseae* [10] are among the key pathogens responsible for avocado anthracnose, significantly impacting avocado fruit quality globally. *C. gloeosporioides* mainly impacts avocado leaves, shoots, flowers, and young fruits, causing fruit drop and rot. For pre-harvest control, carbendazim, thiophanate-methyl, prochloraz, azoxystrobin, pyrimidate, difenoconazole, and propiconazole are primarily used in spraying [13]. As for post-harvest treatment, prochloraz, and imazalil are applied through soaking [14].

This study reports on *Diaporthe phaseolorum* causing avocado fruit rot, and *Colletotrichum fructicola* causing anthracnose in Menglian, Pu’er, Yunnan, China. Fruit rot caused by *Diaporthe phaseolorum* was first reported in China. And fruit anthracnose was caused by *C. fructicola*, severely impacting avocado yield and quality. This study fills the gap on these pathogens and establishes a scientific foundation for targeted prevention and control strategies. These diseases have substantial implications for avocado production, leading to fruit deterioration, drop, and diminished market value. Through accurate pathogen identification, fruit growers can be directed towards employing evidence-based control practices, reducing disease prevalence, enhancing fruit integrity and quality, and safeguarding economic interests.

## 2. Materials and Methods

### 2.1. Sample Collection and Isolation

In November and December 2024, diseased fruits were collected with 4-year-old avocado trees in Menglian, Pu’er, Yunnan, China (99°40′ E, 22°22′ N). A five-point survey was conducted in the orchard, covering a total of 50 trees. From each tree, ten fruits were selected in four directions (east, west, south, and north). A total of 40 samples from various randomly selected fruits were subjected to microscopic examination, and field disease rate was calculated. Hence, the single-spore separation method was employed. Under an anatomical lens and microscope within a laminar flow hood, 10 conidia were extracted from pycnidia. The fruit surfaces were first sterilized with 70% alcohol for 30 s and then dried with absorbent paper. Spores were collected from the diseased fruits using a scalpel and transferred to potato dextrose agar, which was prepared with 200 g of potato, 20 g of glucose, and 17 g of agar per liter. The resulting colonies were then transferred to slant medium (PDA) for preservation and incubated at 25 °C [15]. Strain was stored at Southwest Forestry University. Microscopic measurements were made on cultures cultured for 10 days using microscope (OLYMPUS CX33, OLYMPUS, Tokyo, Japan) for the size and morphological features of conidia, conidiophores, pycnidia, and ascospores (*n* = 50).

### 2.2. DNA Extraction, Polymerase Chain Reaction, and Sequencing

Mycelial plugs with a diameter of 0.5 cm were cultured on PDA for 5 days. Three mycelial plugs were used to inoculate 100 mL of liquid PD (200 g/L potato and 20 g/L dextrose) in conical flasks. These were incubated at 25 °C in the dark with shaking (120 rpm) for about 8 days. The mycelium (approximately 100 mg) was frozen with liquid nitrogen and ground into a powder for subsequent DNA extraction by Genomic DNA kit (Tiangen, DP305, Beijing, China).

Pathogenic isolates and reisolated strains from disease were identified by molecular analysis. Subsequent amplification of different DNA fragments was performed using PCR (Table 1). All primers were synthesized by Sangon Biotech Co., Ltd. (Shanghai, China). PCR system: Premix Taq (Ex Taq Version 2.0 plus dye) 25 μL (Takara. RR902A, Beijing, China), 1 μL DNA sample, 1 μL upstream/downstream primer, up to 50 μL of deionized water. The cycling conditions are as follows: 95 °C for 2 min, 95 °C for 30 s, Tm °C 30 s (Table 1), 72 °C for 2 min, for 35 cycles, 72 °C for 10 min. DNA integrity verification was conducted via 0.8 % agarose gel electrophoresis. The amplified products were analyzed by agarose gel electrophoresis and sequenced using Sanger method by Sangon Biotech Co., Ltd., (Shanghai, China).

The obtained sequences were compared to sequences in GenBank using BLAST (https://blast.ncbi.nlm.nih.gov/). The sequences, including reference sequences from GenBank, were manually aligned using MEGA 11. Phylogenetic relationships were inferred using the maximum-likelihood method with a heuristic search. Bootstrap support values based on 1000 replications were calculated for tree branches [19]. Similarities (%) were calculated using the MegAlign program (Dnastar 7.1). All sequence data from this study have been deposited in GenBank.

### 2.3. Pathogenicity Test

For the pathogenicity test, sixty healthy *P. americana* fruits were used. The fruits were surface-sterilized with 70% alcohol and then inoculated with 5 mL spore suspension (1 × 10^6^ spores/mL). Control branches were inoculated with sterile water. The fruits were placed in a sterilized glass container filled to maintain a relative humidity of 75% and incubated at 26 °C for 15 days. The incubation conditions were designed to simulate the autumn-like climate of Yunnan, which may be necessary for infection. Using the single-spore separation method, the suspected causal agent was re-isolated from symptomatic fruits on PDA plates. The morphological characteristics of the cultured spores were compared with those of spores found in the field.

## 3. Results

### 3.1. Diaporthe Phaseolorum

**Figure 1:**
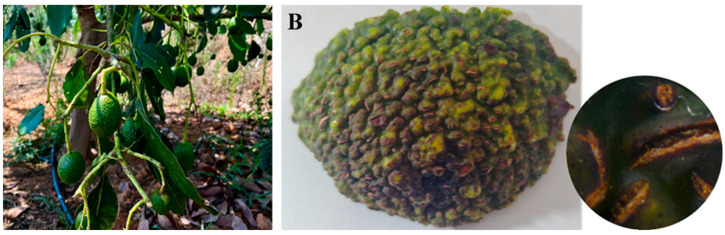
Symptoms of avocado fruit rot in the field: (**A**) Diseased avocado fruits in the field; (**B**) A sample of diseased fruits.

The avocado fruits turn reddish-brown, cracking. Subsequently, the fruits undergo premature maturation, resulting in a soft, black, and decayed state. In high humidity conditions, a brown exudate flows out (Figure 1A,B). The incidence rate was 22% in the field.

#### 3.1.1. Description

**Figure 2:**
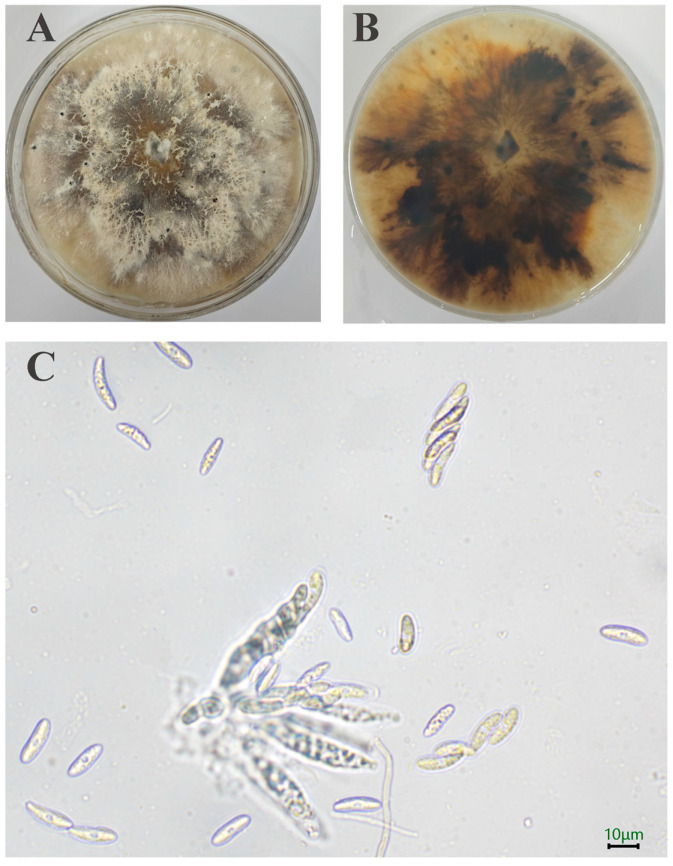
Morphology of *D. phaseolorum*: (**A**,**B**) Colony on PDA after 15 days; (**C**) Asci and Ascospore. The scale = 10 μm.

A total of 10 strains is separated out. Initially, the SWFU20 strain colonies appear white, featuring flocculent and dense hyphae with irregular margins. Subsequently, the central region of the colony transitions to a brown color, with pigmentation gradually deepening and spreading outward. After a 15-day incubation period, minute black spots become evident on the surface of the sample (Figure 2A,B). The ascus is sessile, directly attached to the substrate without a stalk, and exhibits a long clavate morphology, containing 8 ascospores. The ascospores are hyaline, smooth-walled, ellipsoid in shape with apical tapering, and possess a median septum with 3.15–6.24 × 13.03–21.66 μm (n = 50) (Figure 2C).

#### 3.1.2. Phylogenetic Analysis

**Figure 3:**
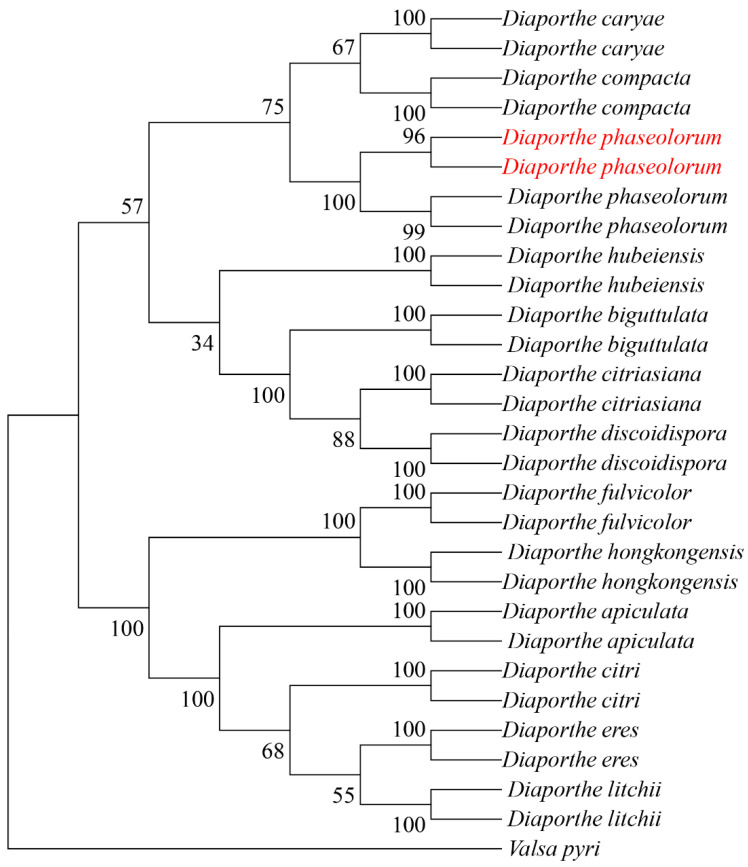
Maximum likelihood tree of *D. phaseolorum* generated from combined *ITS*, *TUB*, and *TEF* sequence data. The values above the branches indicate bootstrap values. Numbers above branches show bootstrap support values inferred from maximum likelihood. Newly obtained sequences in this study are marked in red.

The internal transcribed spacer (*ITS*), *β*-tubulin gene (*TUB*), and translation elongation factor 1-alpha (*TEF1-α*) were amplified using primer pairs ITS1/ITS4, Bt2a/Bt2b, and EF1-728/EF1-986, respectively. BLAST comparison in the NCBI database showed 100%, 99.78%, and 99.70% similarity with reference strains of *D. phaseolorum* KM979757.1, MW595779.1 and KC343905.1, respectively. All sequences were deposited in GenBank under accession numbers PV056040 and PV056041 (*ITS*), PV056051 and PV056052 (*TUB*), and PV056053 and PV056054 (*TEF1-α*). Phylogenetic analysis was performed in MEGA 11 using maximum-likelihood methodology with concatenated sequences. The dataset included 81 reference sequences from 14 taxa retrieved from GenBank (Table 2). The resulting phylogeny supported the placement of our isolates within the *D. phaseolorum* clade (Figure 3).

#### 3.1.3. Pathogenicity Test

**Figure 4:**
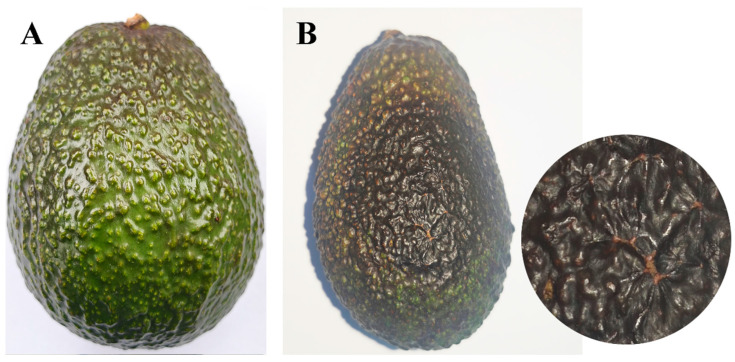
Comparison of avocado fruits mock-inoculated with sterile water versus *D. phaseolorum* at 15 days post-inoculation: (**A**) No symptoms were observed on the healthy control fruit inoculated with water; (**B**) Symptoms of inoculated with *D. phaseolorum* on avocado fruit and enlargement of the image.

To verify pathogenicity, a spore suspension (1 × 10^6^ conidia/mL) was inoculated with 20 attached fruits, and the control was inoculated with ddH_2_O. Subsequently, the inoculated fruits were covered with plastic bags with 70% relative humidity, for 72 h, then incubated at 25 °C. After 15 days, typical rot symptoms, similar to those observed in the field, appeared on inoculated fruits, while no symptoms were observed on the control fruits. Pathogen SWFU21 was reisolated from the site of disease and identified morphologically and molecularly as being consistent with *D. phaseolorum* SWFU20 (Figure 4).

### 3.2. Colletotrichum Fructicola

**Figure 5:**
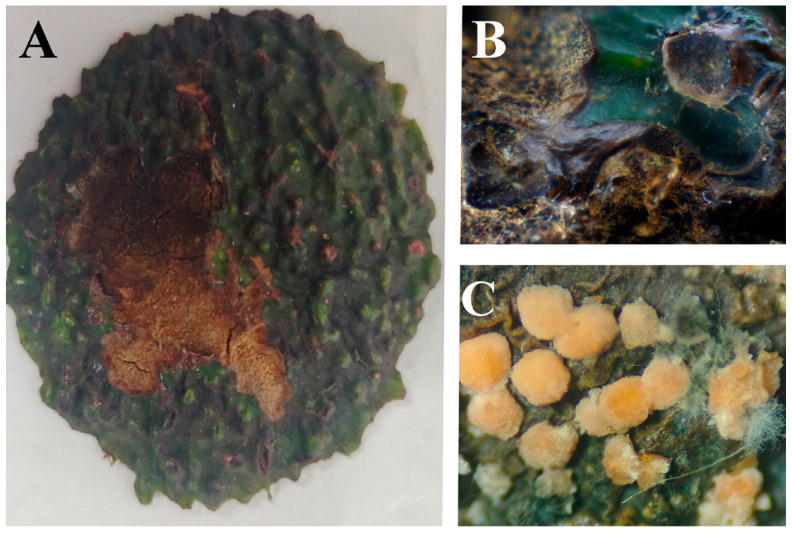
Symptoms of avocado fruit anthracnose in the field: (**A**) Fruit anthracnose; (**B**) Typical symptoms on diseased fruits; (**C**) Mature acervuli.

In the initial stage, lesions exhibit a central depression. As the disease progresses, small black acervuline develop on the lesions. During the intermediate phase, lesions expand rapidly with increasing depression, often accompanied by radial cracking at the center. In advanced stages, the pathogen penetrates the fruit pulp, causing dark green, dry rot of the fleshy tissue. Severe infections lead to progressive deterioration of internal fruit structures. With high humidity conditions, orange conidial masses become evident on the lesion surface (Figure 5). The disease incidence in the field is 15%.

#### 3.2.1. Description

**Figure 6:**
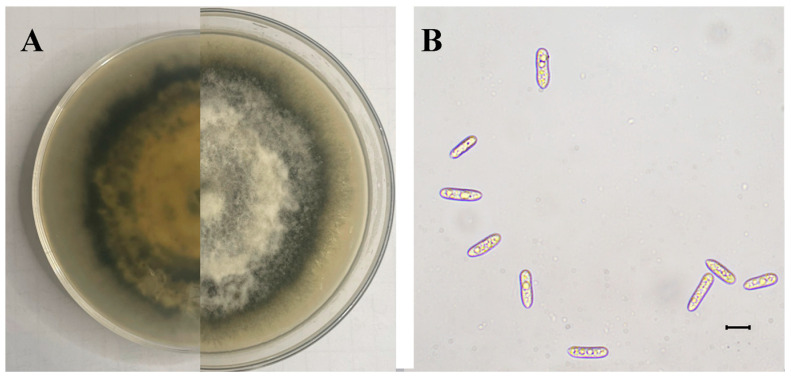
Morphology of *C. fructicola*: (**A**) Colony on PDA after 15 days; (**B**) Conidia. The scale is 10 μm.

The SWFU12 strain was obtained. Colonies initially appeared white before gradually developing gray to dark gray pigmentation. The colony center displayed dark gray coloration that grew lighter towards the periphery, forming a distinct dark gray ring at the outer edge (Figure 6A). Mycelial growth exhibited a tomentose texture with dense, gray aerial mycelium. The conidia were single-cell, oblong-ellipsoid to ellipsoid, and colorless, with dimensions ranging from 12.27 to 20.07 × 2.58–6.87 µm (mean 16.34 × 4.68 µm, n = 50) (Figure 6B).

#### 3.2.2. Phylogenetic Analysis

**Figure 7:**
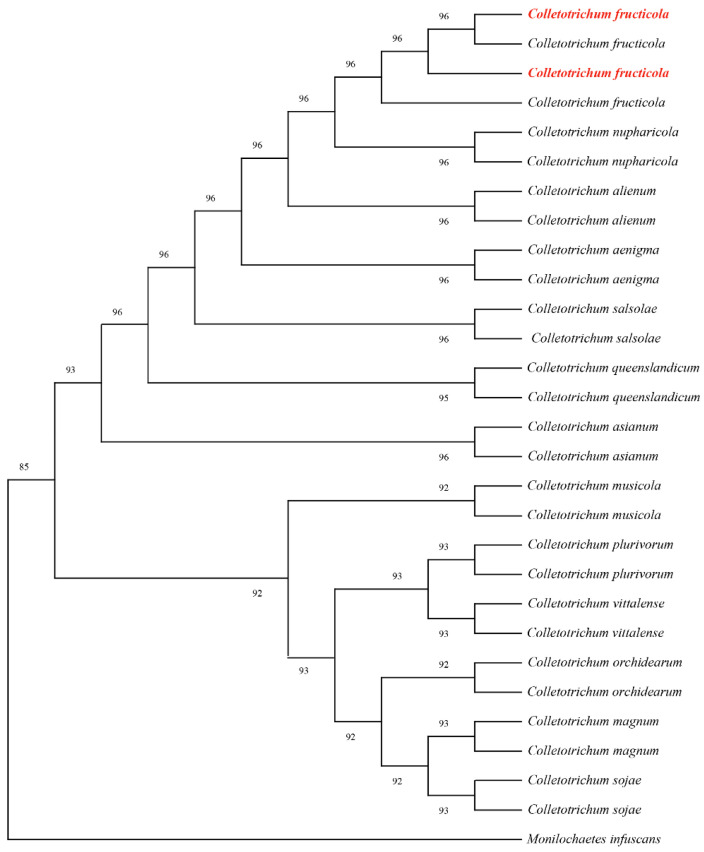
Maximum likelihood tree of *C. fructicola* generated from combined *ITS*, *ACT*, and *GAPDH* sequence data. The values above the branches indicate bootstrap values. The numbers above branches show bootstrap support values inferred from maximum likelihood. Newly obtained sequences in this study are marked in red.

Partial gene sequences of the internal transcribed spacer (*ITS*), actin (*ACT*), and glyceraldehyde-3-phosphate dehydrogenase (*GAPDH*) were amplified using primer pairs ITS1/ITS4, ACT-512/ACT-783, and GDF/GDR, respectively. BLAST analysis revealed 99.81%, 100%, and 100% sequence homology with *C. fructicola* reference sequences OQ652404, OP750663, and KU743270. All sequences were deposited in GenBank accession numbers PQ866913 PQ866914 (*ITS*), PQ876936, PQ876937 (*ACT*), and PQ997936, PQ997937 (*GAPDH*). Phylogenetic analysis was conducted using MEGA 11 with the maximum-likelihood method and concatenated sequences aligned against 81 reference sequences representing 14 taxa from GenBank (Table 3). The resulting phylogeny supported the taxonomic placement within *C. fructicola* clades (Figure 7). Combined morphological and molecular analyses conclusively identified the strain as *C. fructicola*.

#### 3.2.3. Pathogenicity Test

**Figure 8:**
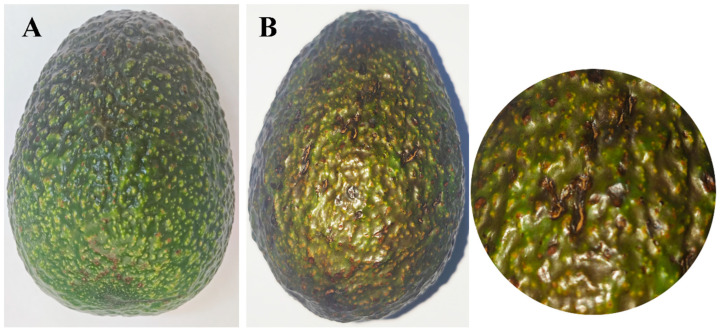
Comparison of avocado fruits mock-inoculated with sterile water versus *C. fructicola* at 15 days post-inoculation: (**A**) No symptoms were observed on the healthy control fruit inoculated with water; (**B**) Symptoms on avocado fruit inoculated with *C. fructicola* and enlargement of the image.

After 15 days post-inoculation, the fruits that had been reinoculated displayed symptoms that were analogous to those that had been observed in the field. However, no symptoms were detected on the control fruits (Figure 8). The strain SWFU13 was reisolated from the re-inoculated fruits, and the morphological and molecular characteristics were consistent with those of *C. fructicola* SWFU12.

## 4. Discussion

In Menglian, Yunnan, avocado fruit rot and anthracnose represent prevalent diseases, and according to our survey, the incidence rate of affected orchards is 15% and 22%, respectively. These pathogens significantly compromise both yield and fruit quality. Based on our experimental observation and previous reports, fruit rot manifests as tissue necrosis and internal decay, resulting in complete loss of edibility. Anthracnose infection produces characteristic surface lesions, impairing visual quality and potentially inducing premature fruit abscission. The combined effects substantially reduce marketability while disrupting normal ripening processes, ultimately affecting flavor development and overall fruit quality.

Anthracnose is caused by multiple *Colletotrichum* sp., resulting in severe yield losses of vegetables [20], fruits, and trees. *C. fructicola* is a phytopathogen with a broad host range that produces melanized appressoria and perithecia [21]. *C. fructicola* was reported to cause anthracnose on *Ziziphus mauritiana* [22], *Arachis hypogaea* [23], *Shine Muscat* [24], *Brassica parachinensis* [11], *Kadsura coccinea* [25], *Rosa chinensis* [26], and so on. Fruit anthracnose was observed on *Zanthoxylum bungeanum* [27], *Prunus avium* [28], *Fragaria xananassa* [29], *Nephelium lappaceum* [30], and *Luffa cylindrica* [31] in China, and leaf spot on *Dalbergia hupeana* [32], *Millettia speciosa* [33], *Manglietia decidua* [34], *Illicium verum* [35], *Smilax glabra* [36], *Prunus sibirica* [37] in China. The identification of *C. fructicola* can be confirmed by the internal transcribed spacer and three partial gene regions (*β*-tubulin, glyceraldehyde-3-phosphate dehydrogenase, and actin) [38]. For a long time, the prevention and control of *C. fructicola* has been conducted mainly using fungicides such as pyraclostrobin, procymidone, prochloraz, and fludioxonil [39]. Avocado plantations in China utilize this type of fungicide for the prevention and control of *C. fructicola* in the future. It is worth noting that *C. fructicola* has been found to be resistant to fludioxonil [39]. Therefore, it is very necessary to prevent and control the diseases caused by *C. fructicola* through biological interaction in the future.

*D. phaseolorum* is a widely distributed phytopathogenic fungus that causes root and stem rot in soybean worldwide [40], particularly in Europe and the Americas. As a non-host-selective pathogen [41], it can damage a wide range of cash crops. This pathogen causes stem gray spot in *Hylocereus polyrhizus* [42], also postharvest rot in *Actinidia* sp. [43], *Helianthus annuus* [44], *Euphorbia neriifolia* var. cristata [45], and ulcers in *Vaccinium* spp. [46]. Additionally, it causes leaf spot disease in *Bletilla striata* [47] and wilt in *Melia dubia* [48]. Symptoms of these diseases typically include brown to reddish-brown necrotic spots on stalks and crinkling and cracking of seed shells [49]. These symptoms are similar to those we observed on avocado fruits. The fungus has an extremely wide distribution, covering almost all regions except the boreal zone. To accurately identify *D. phaseolorum*, researchers commonly use sequences of conserved regions such as the internal transcribed spacer (*ITS*) region of the ribosome, part of the calmodulin gene (*CAL*), the DNA-cleaving enzyme gene (*APN2*), the histone H3 gene (*HIS3*), the translation elongation factor-1alpha gene (*TEF1*), and the *β*-microtubulin gene (*TUB2*). Previous studies have shown that *D. phaseolorum* colonizes the plant body via ergosterol [50]. Current control measures against this pathogen mainly include direct control using quinolines [25] and *Bacillus* spp. [51] or indirect control by increasing plant resistance [27]. We can try these methods to address the problem of avocado being infected by *D. phaseolorum* in China. However, the effectiveness of these methods in preventing and controlling this pathogen in other species is not satisfactory. Therefore, developing more effective control technologies for *D. phaseolorum* to ensure the sustainable development of the avocado industry remains an urgent issue.

## 5. Conclusions

The pathogen causing avocado fruit rot and fruit anthracnose in the Menglian of Yunnan were systematically identified as *D. phaseolorum* and *C. fructicola*, respectively. This discovery addresses a significant gap in the understanding of avocado fruit diseases and establishes a robust scientific foundation for subsequent disease prevention and control measures. Fruit rot and fruit anthracnose have a substantial impact on avocado yield and quality, leading to deformities, rot, shedding, and diminished market value. This study reports on these two pathogens for the first time, which will assist farmers in more accurately identifying pathogens, thus enabling them to implement targeted prevention and control measures, reduce the incidence rate of diseases, enhance fruit quality, and protect the economic interests of growers. Moreover, this research supports the development of monitoring and early warning systems for both diseases, enabling growers to implement timely interventions to mitigate disease-related losses. The study facilitates the exchange of knowledge on avocado disease prevention and control practices among different regions, thereby advancing the avocado industry in China. Future research will delve into the biological characteristics, pathogenic mechanisms, and interactions with environmental factors of these pathogens to underpin the development of more effective and environmentally sustainable disease prevention and control strategies, supporting the sustainable growth of the avocado industry in China. This study did not screen for preventive and therapeutic agents or methods specifically targeting these two pathogens. Future research can build on this foundation to explore the topic further.

## Figures and Tables

**Table 1:** Primer sequences.

Region	Primer	Forward/Reverse	Tm (°C)	References
*ITS*	ITS1 ITS4	F: TCCGTAGGTGAACCTGCGG R: TCCTCCGCTTATTGATATGC	50	[16]
*GAPDH*	GDF GDR	F: GCCGTCAACGACCCCTTCATTGA R: GGGTGGAGTCGTACTTGAGCATGT	62	[8]
*ACT*	ACT-512 ACT-783	F: ATGTGCAAGGCCGGTTTCGC R: TACGAGTCCTTCTGGCCCAT	60	[8]
*TUB*	Bt2a Bt2b	F: GGTAACCAAATCGGTGCTGCTTTC R: ACCCTCAGTGTAGTGACCCTTGGC	61	[17]
*TEF1-α*	EF1-728 EF1-986	F: CATCGAGAAGTTCGAGAAGG R: TACTTGAAGGAACCCTTACC	52	[18]

**Table 2:** GenBank accession numbers used for phylogenetic analysis of *D. phaseolorum*.

Species and Strain	Locality, Host/Substrate	GenBank Accession Numbers		
		*ITS*	*TUB*	*TEF1-α*
***Diaporthe caryae***				
ZJUE0276	China, *Citrus reticulata*	ON035561	ON221770	ON049537
ZJUE0281	China, *Citrus reticulata*	ON035562	ON221771	ON049538
***Diaporthe compacta***				
ZJUE0269	China, *C. unshiu*	ON035573	ON221782	ON049549
ZJUE0270	China, *C. unshiu*	ON035574	ON221783	ON049550
***Diaporthe phaseolorum***				
**SWFU20**	**China, *P. americana***	**PV056040**	**PV056051**	**PV056053**
**SWFU21**	**China, *P. americana***	**PV056041**	**PV056052**	**PV056054**
Ar2 CBS 116019	USA, *Arctium lappa*; USA, *Caperonia palustris*	HM347705	KC344143	HM347679
PS03 CBS 116020	Croatia, *Glycine max*; USA, *Symphyotrichum subulatum*	HM347702	KC344144	HM347670
***Diaporthe hubeiensis***				
ZJUE0393	China, *C. reticulata*	OP218147	OP265666	OP265602
ZJUE0400	China, *C. sinensis*	OP218148	OP265667	OP265603
***Diaporthe biguttulata***				
ZJUE0369	China, *C. sinensis*	OP218118	OP265637	OP265573
ZJUE0398	China, *C. reticulata*	OP218119	OP265638	OP265574
***Diaporthe citriasiana***				
ZJUE0217	China, *C. hybrid*; *C. reticulata*; *C. reticulata*	ON035571	ON221780	ON049547
ZJUE0286	China, *C. unshiu*	ON035572	ON221781	ON049548
***Diaporthe discoidispora***				
ZJUE0384	China, *C. sinensis* cv. Newhall	OP218131	OP265650	OP265586
ZJUE0410	China, *C. reticulata*	OP218132	OP265651	OP265587
***Diaporthe fulvicolor***				
ZJUE0310	China, *C. limon*	ON035588	ON221797	ON049564
ZJUE0311	China, *C. limon*	ON035589	ON221798	ON049565
***Diaporthe hongkongensis***				
ZJUE0289	China, *C. sinensis*	ON035592	ON221801	ON049568
ZJUE0290	China, *C. sinensis*	ON035593	ON221802	ON049569
***Diaporthe apiculate***				
ZJUE0357	China, *C. reticulata*	OP218106	OP265625	OP265561
ZJUE0358	China, *C. reticulata*	OP218107	OP265626	OP265562
***Diaporthe citri***				
ZJUE0154	China, *C. unshiu*	ON035564	ON221773	ON049540
ZJUE0223	China, *C. unshiu*	ON035565	ON221774	ON049541
***Diaporthe eres***				
ZJUE0148	China, *C. unshiu*	ON035577	ON221786	ON049553
ZJUE0149	China, *C. unshiu*	ON035578	ON221787	ON049554
***Diaporthe litchi***				
ZJUE033 ZJUE0344	China, *C. maxima*; Unknown	OR160298	OR178895	OR178892
ZJUE0341	China, *C. maxima*	OR160299	OR178896	OR178893
***Valsa pyri***				
G1H	China, *Pyrus* sp.	OR122731	OR876852	OR876853

**Table 3:** GenBank accession numbers used for phylogenetic analysis of *C. fructicola*.

Species and Strain	Locality, Host/Substrate	GenBank Accession Numbers		
		*ITS*	*ACT*	*GAPDH*
***Colletotrichum fructicola***				
**SWFU12**	**China, *Persea americana***	**PQ866913**	**PQ876936**	**PQ997936**
**SWFU13**	**China, *Persea americana***	**PQ866914**	**PQ876937**	**PQ997937**
PXSL_X43 PCZJJD27 G62A	China, *Camellia oleifera*; China, *Carya illinoensis*; Puerto Rico, *Dioscorea alata*	OQ652404	OP750663	KU743270
HNPMCs_3_1A PCZJJD26 CgUC21	Myanmar, *Camellia sinensis*; China, Unknow; Philippines, *Mangifera indica*	MZ348542	OP750697	MT407955
***Colletotrichum nupharicola***				
C1275.25	USA, *Nuphar polysepala*	JX010187	JX009437	JX009972
C1275.24	USA, *Nuphar polysepala*	JX010189	JX009486	JX009936
***Colletotrichum alienum***				
C824	New Zealand, *Malus domestica*	JX010251	JX009572	JX010028
C1194.22	New Zealand, *Persea americana*	JX010246	JX009552	JX009959
***Colletotrichum aenigma***				
C1253.4	Israel, *Persea americana*	JX010244	JX009443	JX010044
C1256.6	Japan, *Pyrus pyrifolia*	JX010243	JX009519	JX009913
***Colletotrichum salsolae***				
C1314	Hungary, *Salsola tragus*	JX010242	JX009562	JX009916
C1257.6	Hungary, *Glycine max*	JX010241	JX009559	JX009917
***Colletotrichum queenslandicum***				
C956.1	Fiji, *Coffea* sp.	JX010185	JX009490	JX010036
ICMP 1778	Australia, *Carica papaya*	JX010276	JX009447	JX009934
***Colletotrichum asianum***				
C1315.4	Thailand, *Coffea arabica*	JX010196	JX009584	JX010053
C1187	Australia, *Mangifera indica*	JX010192	JX009576	JX009915
***Colletotrichum musicola***				
CBS 132885	Unknow	MG600736	MG600942	MG600798
CBS 127557	Unknow	MG600737	MG600943	MG600799
***Colletotrichum plurivorum***				
CBS 125474	Unknow	MG600718	MG600925	MG600781
CBS 132443	Unknow	MG600719	MG600926	MG600782
***Colletotrichum vittalense***				
CBS 12625	Unknow	MG600735	MG600941	MG600797
CBS 18182	Unknow	MG600734	MG600940	MG600796
***Colletotrichum orchidearum***				
CBS 135131	Unknow	MG600738	MG600944	MG600800
CBS 136877	Unknow	MG600739	MG600945	MG600801
***Colletotrichum magnum***				
CBS 51997	Unknow	MG600769	MG600973	MG600829
IMI391662	Unknow	MG600771	MG600975	MG600831
***Colletotrichum sojae***				
ATCC 62257	Unknow	MG600749	MG600954	MG600810
CBS 128510	Unknow	MG600751	MG600956	MG600812
***Monilochaetes infuscans***				
CBS 869.96	Unknow	JQ005780	JQ005843	JX546612

## Data Availability

The original contributions presented in this study are included in the article. Further inquiries can be directed to the corresponding authors.

## References

[1] Araújo R.G., Rodriguez-Jasso R.M., Ruiz H.A., Pintado M.M.E., Aguilar C.N. (2018). Avocado by-products: Nutritional and functional properties. Trends Food Sci. Technol..

[2] Sommaruga R., Eldridge H.M. (2021). Avocado production: Water footprint and socio-economic implications. EuroChoices.

[3] Perkins M.L., Joyce D.C., Coates L.M. (2019). Possible contribution of impact injury at harvest to anthracnose expression in ripening avocado: A review. Sci. Hortic..

[4] Moller H., Slippers B., van den Berg N. (2025). Branch canker battles: Understanding and managing the Botryosphaeriaceae in avocado. Phytoparasitica.

[5] Kebede M., Belay A. (2019). Fungi associated with post-harvest avocado fruit rot at jimma town, southwestern Ethiopia. J. Plant Pathol. Microbiol..

[6] Wogu M.D. (2014). Microorganisms associated with the spoilage of avocado pear, *Persea americana* fruits. Afrrev Stech.

[7] Shi T.F., Pan T.T., Guo M.T. (2022). First isolation and identification of *Neopestalotiopsis clavispora* causing postharvest rot of Rosa sterilis and its control with methyl jasmonate and calcium chloride. Horticulturae.

[8] Weir B.S., Johnston P.R., Damm U. (2012). The *Colletotrichum gloeosporioides* species complex. Stud. Mycol..

[9] Bustamante M.I., Osorio-Navarro C., Fernández Y., Bourret T.B., Zamorano A., Henríquez-Sáez J.L. (2022). First record of *Colletotrichum anthrisci* causing anthracnose on avocado fruits in Chile. Pathogens.

[10] Hofer K.M., Braithwaite M., Braithwaite L.J., Sorensen S., Siebert B., Pather V., Goudie L., Williamson L., Alexander B.J.R., Toome-Heller M. (2021). First Report of *Colletotrichum fructicola*, *C. perseae*, and *C. siamense* Causing Anthracnose Disease of Avocado (Persea americana) in New Zealand. Plant Dis..

[11] Yu L., Lan G.B., Yang Y.G., Tang Y.F., Li Z.G., She X.M., He Z.F. (2022). First report of anthracnose caused by *Colletotrichum fructicola* on *Brassica parachinensis* in China. Crop Prot..

[12] Li S., Liu Z., Zhang W. (2022). First Report of Anthracnose Disease on Avocado (*Persea americana*) Caused by *Colletotrichum fructicola* in China. Plant Dis..

[13] Fischer I.H., Tozze Junior H.J., de Arruda M.C., Massola Junior N.S. (2011). Postharvest of ‘Fuerte’ and ‘Hass’ avocados: Physical and chemical characteristics, damages and control of diseases. Semin.-Cienc. Agrar..

[14] Moreira V., Carbone M.J., Ferronato B., Gonzalez-Barrios P., Alaniz S., Mondino P. (2025). Role of fungicides to control blossom blight and fruit rot the main symptoms of olive anthracnose in Uruguay. Int. J. Pest Manag..

[15] Chen J., Liu X.F., Jia H.Q., Zhu W.B. (2018). First report of leaf-spot disease caused by *Sphaeropsis visci* on Asian mistletoe [*Viscum coloratum* (Kom. ) Nakai] in China. J. For. Res..

[16] White T.J., Bruns T., Lee S., Taylor J. (1990). Amplification and direct sequencing of fungal ribosomal RNA Genes for phylogenetics. PCR Protocols Guide Methods Appl..

[17] Glass N.L., Donaldson G.C. (1995). Development of primer sets designed for use with the PCR to amplify conserved genes from filamentous ascomycetes. Appl. Environ. Microb..

[18] Carbone I., Kohn L.M. (1999). A Method for Designing Primer Sets for Speciation Studies in Filamentous Ascomycetes. Mycologia.

[19] Hillis D., Bull J. (1993). An Empirical Test of Bootstrapping as a Method for Assessing Confidence in Phylogenetic Analysis. Syst. Biol..

[20] Cao L.X., Shi K.L., Liu Y.Y., Xie X.N., Sun X.Z., Dong W.T., Wang C.Y., Ma L.S. (2024). Identification of specific genes as molecular markers for rapid and accurate detection of oil-tea *Camellia* anthracnose pathogen *Colletotrichum fructicola* in China. Front. Microbiol..

[21] Liang X.F., Li B.X., Zhao X.M., Yao L.Q., Kong Y.Y., Liu W.K., Zhang R., Sun G.Y. (2022). 1, 8-Dihydroxynaphthalene melanin biosynthesis in *Colletotrichum fructicola* is developmentally regulated and requires the cooperative function of two putative zinc finger transcription factors. Phytopathology.

[22] Duan C.H., Chen G.Y. (2022). First report of *Colletotrichum fructicola* causing anthracnose on Indian Jujube (*Ziziphus mauritiana*) in Taiwan. Plant Dis..

[23] Gong J.L., Sun D.L., Bian N.F., Wang R.Q., Wang X., Wang X.J. (2023). First report of *Colletotrichum fructicola* causing anthracnose on peanut (*Arachis hypogaea*) in China. Plant Dis..

[24] Lim Y.S., Hassan O., Chang T. (2020). First report of anthracnose of shine muscat caused by *Colletotrichum fructicola* in Korea. Mycobiology.

[25] Wang T., Ren Y.L., Zhao J.Y., Jiang Y.J., Tang J., Liu Y., Liu C., Wang J., Ji X.L., Wang M.Y. (2022). Identification of pathogens and laboratory activity test of kiwifruit rot disease in Guizhou Province, China. J. Chem..

[26] Du L., Du C.Y., Ding C.Y. (2023). First Report of *Colletotrichum fructicola* Causing Anthracnose on Rosa chinensis in China. Plant Dis..

[27] Yang X., Lu L.L., Wang Y., Sun Y., Geng Z.J., Wei B.Y., Tang G.H., Li P.Q. (2024). First report of *Colletotrichum fructicola* causing fruit anthracnose on Chinese prickly ash (*Zanthoxylum bungeanum*) in China. Plant Dis..

[28] Tang Z.Y., Lou J., He L.Q., Wang Q.D., Chen L.H., Zhong X.T., Wu C.F., Zhang L.Q., Wang Z.Q. (2022). First report of *Colletotrichum fructicola* causing anthracnose on cherry (*Prunus avium*) in China. Plant Dis..

[29] Zou X.H., Guo R., Zhang L.Q., Duan K., Gao Q.H. (2018). Identification of FaNBS-encoding genes responsive to *Colletotrichum fructicola* infection in strawberry (*Fragaria× ananassa* Duchase). Australas. Plant Path..

[30] Serrato-Diaz L., Rivera-Vargas L., Goenaga R., Navarro E., French-Monar R. (2017). First report of *Colletotrichum fructicola* and *C. queenslandicum* causing fruit rot of rambutan (*Nephelium lappaceum*). Plant Dis..

[31] Li P., Zhu J.Z., Li X.G., Zhong J. (2022). Identification and characterization of *Colletotrichum fructicola* and *Colletotrichum siamense* causing anthracnose on luffa sponge gourd in China. Plants.

[32] Zhou Y., Ye R., Ying Q., Zhang Y., Zhang L.P. (2022). First report of leaf spot caused by *Colletotrichum fructicola* on *Dalbergia hupeana* in China. Plant Dis..

[33] Shu J., Ning P., Guo T.X., Tang L.H., Huang S.P., Li Q.L., Mo J.Y., Yu Z.H., Hsiang T. (2020). First report of leaf spot caused by *Colletotrichum fructicola* on *Callerya speciosa* (*Millettia speciosa*) in Guangxi, China. Plant Dis..

[34] Liu H.H., Zhu K.X., Li C.Z., Wei C.H., Luan F.G., Li D., Song Q.N. (2023). First Report of Anthracnose Leaf Spot Caused by *Colletotrichum fructicola* on *Manglietia decidua* (Magnoliaceae) in China. Plant Dis..

[35] Zhao J., Yu Z.H., Li Q.L., Tang L.H., Guo T.X., Huang S.P., Mo J.Y., Hsiang T. (2022). Leaf spot caused by *Colletotrichum fructicola* on star anise (*Illicium verum*) in China. Plant Dis..

[36] Liu Z., Wang H.Y., Lin S.Y., Guo J.Y., Shi Y.P., Gao Q., Zhou H. (2024). First Report of *Colletotrichum fructicola* Causing Leaf Spot on Smilax glabra in China. Plant Dis..

[37] Han S., Xu X., Jiang Y.R., Zheng Z.L., Yuan H., Li S.J., Liu Y.G., Lin T.T., Qiao T.M., Yang C.L. (2023). *Colletotrichum fructicola*, Causal Agent of Shot-Hole Symptoms on Leaves of *Prunus sibirica* in China. Plant Dis..

[38] Negi N., Ramkrishna, Banerjee S., Meena R.K., Bhandari M.S., Pandey S. (2025). Morphology and phylogeny revealed *Colletotrichum fructicola* causing new disease symptoms on *Eucalyptus* in India. J. Plant Pathol..

[39] Usman H.M., Tan Q., Karim M.M., Adnan M., Yin W.X., Zhu F.X., Luo C.X. (2021). Sensitivity of *Colletotrichum fructicola* and *Colletotrichum siamense* of peach in China to multiple classes of fungicides and characterization of pyraclostrobin-resistant isolates. Plant Dis..

[40] Pioli R.N., Morandi E.N., Martínez M.C., Lucca F., Tozzini A., Bisaro V., Hopp H.E. (2003). Morphologic, molecular, and pathogenic characterization of *Diaporthe phaseolorum* variability in the core soybean-producing area of Argentina. Phytopathology.

[41] Gi S., Kim W., Yang K.Y. (2022). Emergence of multiple *Diaporthe* species causing kiwifruit rot and occurrence of resistance to a methyl benzimidazole carbamate fungicide in South Korea. Crop Prot..

[42] Huda-Shakirah A.R., Kee Y.J., Wong K.L., Zakaria L., Mohd M.H. (2021). *Diaporthe* species causing stem gray blight of red-fleshed dragon fruit (*Hylocereus polyrhizus*) in Malaysia. Sci. Rep..

[43] Kai K., Bi W.L., Sui Y., Hua C.Y., Liu Y.S., Zhang D.F. (2020). Curcumin inhibits *Diaporthe phaseolorum* and reduces postharvest decay in kiwifruit. Sci. Hortic..

[44] Gomzhina M.M., Gannibal P.B. (2022). Diaporthe species infecting sunflower (*Helianthus annuus*) in Russia, with the description of two new species. Mycologia.

[45] Li P.P., Cao Z.Y., Liu N., Ma S.X., Dai D.Q., Dong J.G., Ye J. (2017). First Report of *Diaporthe phaseolorum* causing stem canker of *Euphorbia neriifolia* var. *cristata* in China. Plant Dis..

[46] Latorre B.A., Torres R. (2011). *Diaporthe*/*Phomopsis* complex associated with stem cankers of blueberry in Chile. Phytopathology.

[47] Hou X.M., Zheng M.H., Ao J.J., Wang H.Y., Peng L.L., Zhou H. (2022). Occurrence of Leaf Spot Caused by *Diaporthe phaseolorum* on Bletilla striata in China. Plant Dis..

[48] Krupalini V., Janardhana G.R. (2024). *Diaporthe phaseolorum* causing dieback disease on *Melia dubia* cav. in Karnataka state (India). Arch. Microbiol..

[49] Mena E., Jorajuría M., Gurmendez J., Grijalba P., Ponce de León I. (2025). Multiplex qPCR Assay for Detection and Relative Quantification of *Diaporthe aspalathi*, *D. caulivora*, *D. longicolla* and *D. miriciae* in Soybean Tissues. Plant Pathol..

[50] Xue H.Q., Upchurch R.G., Kwanyuen P. (2006). Ergosterol as a Quantifiable Biomass Marker for *Diaporthe haseolorum* and *Cercospora kikuchi*. Plant Dis..

[51] Miljakovic D., Marinkovic J., Tamindzic G., Bordevic V., Ignjatov M., Milosevic D., Nikolic Z. (2022). Effect of plant growth promoting *Bacillus* spp. on germination and seedling growth of soybean. Legume Res.-Int. J..

